# Thermochemical Transformation of Calcium during Biomass Burning and the Effects on Postfire Aqueous Dissolution of Macronutrients

**DOI:** 10.1021/acs.est.4c04820

**Published:** 2024-09-18

**Authors:** Rixiang Huang, Sarah Nicholas, Zheng Wei

**Affiliations:** † Department of Environmental and Sustainable Engineering, University at Albany, 1400 Washington Ave, Albany, New York 12222, United States; ‡ National Synchrotron Light Source II, Brookhaven National Laboratory, Upton, New York 11793, United States; § Department of Chemistry, University at Albany, 1400 Washington Ave, Albany, New York 12222, United States

**Keywords:** wildland fire, fire ash, thermochemistry, macronutrient, elemental cycling, speciation, X-ray absorption spectroscopy

## Abstract

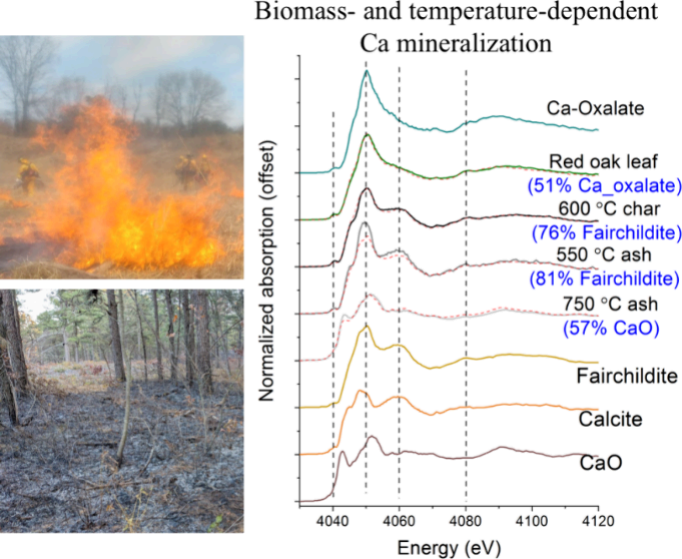

Calcium is commonly the most abundant element in fire residues and its speciation largely determines the geochemical properties of fire residues and their effects on postfire soil chemistry. To explore the effects of biomass composition and fire conditions on ash Ca speciation, this study characterizes the speciation of Ca in charcoal and ash samples that were derived from different plant compartments and thermal conditions, using Ca K-edge X-ray absorption near edge spectroscopy. Results showed that biomass contains abundant organic Ca complexes, which were mineralized into fairchildite and calcite after heating at 450 to 600 °C and then CaO, as temperature increased to 750 °C. Apatite could be an abundant Ca species in fire residues if the Ca/P molar ratio of the biomass is small (<2). The mineralization of organic Ca to the identified Ca minerals during burning was negligibly affected by the oxygen level. Calcium speciation in prescribed fire residues resembled that of biomass ash burned at 550 °C with similar Ca/P molar ratios. Batch experiments showed that macronutrients (Ca, Mg, K, and P) were differentially released, as a result of different solubility of minerals in ashes and reprecipitation of minerals. The aqueous solubility of Ca, Mg, and P decreased as pH increased from 5 to 9, while K showed no pH dependency and was almost completely soluble. Results from this study improve our understanding of the chemistry of fire residues and their geochemical behaviors, which can help evaluate the impact of fire on postfire soil properties and macronutrient cycling.

## Abstract 2

This study quantitatively speciated Ca in fire residues and the results revealed the interplay between biomass composition and fire thermal conditions in controlling Ca speciation and aqueous dissolution of macronutrients.

## 1 Introduction

Fire is a common and possibly the most pervasive disturbance to most terrestrial ecosystems and under climate change their fire regime may experience changes, affecting ecosystem health and succession.^1^ The cycling of macronutrients is among the many ecosystem processes affected by fires. For many nonvolatile macronutrients such as calcium (Ca) and phosphorus (P), internal recycling (the migration within the soil-plant system) represents a major component of their cycles.^2,3^ The burning of aboveground biomass is expected to disturb this internal recycling and the overall cycle of the macronutrients in the ecosystem. Specifically, biomass burning significantly changes the physicochemical forms of the aboveground nutrient pools, which subsequently may affect the pathways and fluxes of nutrients returning to soils.^4^ Therefore, to evaluate the impacts of fires on nutrient cycling, it is important to understand how these macronutrients transform during a fire and their chemical forms in the burned residues. Calcium (Ca) commonly is the most abundant metal macronutrient in plants (0.1–5% of dry mass), serving various physiological functions in plants (e.g., structural component, counterion, and intracellular messenger).^5^ The content and stoichiometry of Ca in plants depend on factors such as plant genotype, compartment, growth stage, and nutrient status.^6,7^ Calcium released from organic matter mineralization can affect soil properties and participates in important soil processes, such as modulation of soil physical structure^8,9^ and solution chemistry, being a significant Ca source for plant uptake^10^ and affecting litter decomposition and organic matter stabilization.^11,12^ Because of the pedogenic and ecological significance of Ca and the contribution of internal recycling of biomass Ca, it is important to explore the effects of fire on Ca cycling, specifically, the cycling of Ca in fire-burned residues.

Calcium exists abundantly as organic complexes (e.g., oxalates) in plants, whose abundance varies among plant parts.^13^ Organic complexes are a significant Ca pool in litter and mineral soils^14^ and the organic Ca is gradually mineralized in soils (e.g., forming carbonates or bonding to clay minerals).^15^ Biomass burning thoroughly mineralizes various elements, forming various inorganic minerals (including Ca). Numerous studies have characterized the composition and mineral phases of ashes from fire, laboratory burning, and energy production with biomass using X-ray diffraction (XRD), and the Ca minerals commonly identified are carbonates (calcite and fairchildite), phosphates (apatite), sulfates (anhydrite and gypsum), and oxides (lime).^16,17^ Despite the above-mentioned progress, a quantitative understanding of the thermochemistry of Ca during vegetation fire remains lacking. First, although common Ca minerals presented in ash have been identified using XRD,^16^ the exact speciation of Ca in ash is yet to be quantitatively determined because XRD is not a sensitive tool to identify and quantify both amorphous and crystalline minerals that exist at relatively low abundances. In particular, contents and species of Ca differ broadly among fuel biomass sources (i.e., plant species, compartments, and age),^5^ variation in ash Ca speciation is thus expected. Second, fire thermal conditions are highly variable, producing ash and charcoal with temperature-dependent properties such as C aromaticity for charcoal.^18^ How Ca speciation evolves during fires and the impacts of burning conditions are not well understood. Therefore, it is necessary to obtain quantitative Ca speciation data to elucidate the thermochemistry of Ca during fire.

The lack of a detailed understanding of macronutrient chemistry in fire residues also impedes the prediction of postfire macronutrient cycling and evaluation of fire disturbance. Although extensive studies have characterized soil nutrient content and availability following fires,^19−21^ the mechanism of macronutrient recycling into soils is not well understood, particularly considering the physicochemical heterogeneity and variability of fire residues. A few studies have characterized aqueous solubilization of macronutrients in fire residues and evaluated the effects of vegetation or fire severity, while variations between samples were observed and a mechanistic understanding remains lacking.^22,23^ For example, P solubilization (in 0.5 M NaHCO_3_) was found to differ significantly among plant ashes and depend on calcite content (determined by thermogravimetric analysis), while the mechanism was unknown.^24^ Therefore, it is necessary to characterize the aqueous solubilization behaviors of Ca and other macronutrients and their relation to their chemistry in fire residues.

In this work, we (1) first explored Ca thermochemistry by characterizing Ca speciation in raw biomass, charcoal, and ash prepared under controlled laboratory conditions, (2) characterized Ca speciation in prescribed fire residues, and (3) characterized the solubility of Ca and other macronutrients and explore the aqueous solubilization mechanisms. We hypothesized that fire temperature and elemental stoichiometry, particularly the molar ratios of Ca to P and potassium (K), primarily regulate Ca speciation in fire-burned residues. Speciation of Ca was quantified primarily by Ca K-edge X-ray absorption spectroscopy, which has been the main tool to nondestructively speciate Ca in environmental samples such as coal and soil, which yields informative results.^15,25^ We also hypothesized that fire ash is unstable following hydration and will experience extensive transformation that affects the solubility of other macro-nutrients such as P, K, and Mg. Therefore, ash transformation following precipitation was simulated using batch equilibrium experiments, and dissolution of Ca and other macro-nutrients were measured. The interplay between biomass elemental composition and thermal conditions in controlling Ca speciation transformation was demonstrated using biomass samples that vary broadly in elemental stoichiometry (e.g., Ca/P and Ca/K). The biomass was heated under highly controlled conditions: (1) complete burning in air and (2) pyrolysis in the absence of oxygen, both of which at different temperatures. The use of known biomass with uniform composition and controlled heating conditions enables the quantitative evaluation of the effects of these factors, which can hardly be realized with real fire ash samples. However, prescribed fire residues were also collected and characterized to validate findings from lab-prepared samples.

## 2 Materials and Methods

### 2.1 Sample Collection

Because our main goal is to evaluate the impacts of biomass feedstock composition (but not to represent a specific ecosystem), we deliberately selected a few biomass types that differ greatly in elemental stoichiometry (molar ratio of Ca to P and K), from a range of plant species and parts that were previously characterized.^26^ The biomass included the cone and needle of Norway spruce (*Picea abies*) and the leaf, wood, and bark of red oak (*Quercus rubra*), which were collected from the Five Rivers Environmental Education Center and a residential neighborhood at Albany, NY. The leaf and fruit were senesced biomass collected and pooled from at least three different spots, and the wood and bark were collected from living plants during tree cutting. The biomass samples were dried in an oven at 70 °C for at least 3 days until no further weight change and then were pulverized using a blender prior to thermal treatments.

Real fire residues were collected from prescribed fires at a local landscape preserve (Albany Pine Bush Preserve, NY, USA) that extensively uses prescribed fires. Prescribed fires at two sites with different vegetation covers were selected, a site with only grass (6/2022) and another site with mainly pitch pine and oaks (11/2023), and the samples were labeled as APBP_grass and APBP_wood, respectively. Considering the fact that prescribed fire residues are generally formed from mixed fuel sources and experience dynamic thermal conditions, the samples complement the laboratory burning samples for understanding the interaction between biomass composition and fire conditions. The fire residue samples were collected the same day of prescribed fires using scoops and consisted of a mixture of complete and incomplete combustion products. The ash was carefully collected and free of soil contamination. The ash was directly used without further processing (except for grinding for spectroscopic analysis).

### 2.2 Pyrolysis and Complete Burning of Biomass

The incomplete and complete burning of biomass during fire was simulated by pyrolysis in the absence of O_2_ and heating in air using a benchtop furnace, respectively. For pyrolysis, the pulverized biomass was tightly packed into 20 mL glass vials and buried in a sand buckle (15 cm below the surface). The sand buckle was heated in a furnace for 6 h (soaking time) at 450 and 600 °C. For complete burning in air, the dry pulverized biomass was placed in a ceramic bowl and heated in air at 550 and 750 °C. Fire temperature is highly variable and dynamic, ranging between 200 and 1100 °C, depending on fire severity and spatial position (high temperatures generally present in the flames).^18,27^ The selected temperatures represent the normal range of wildland fire thermal conditions. The derived samples were labeled as abbreviation + temperature. The abbreviations for the cone and needle of Norway spruce were SC and SN, respectively. The abbreviations for the leaf, wood, and bark of red oak were: ROL, ROW, and ROB, respectively. For ashes of red oak leaf that were burned at 550 and 750 °C, the samples were labeled ROL550 and ROL750, respectively.

### 2.3 Characterization of Physical and Chemical Properties of Ash and Charcoal

Contents of macronutrients in the complete burned ash samples and the prescribed fire samples were analyzed by total digestion of the samples (using Aqua Regia) and measurement by an inductively coupled plasma optical emission spectrophotometer (ICP-OES). The mineralogy of the ash samples was characterized by X-ray diffraction (XRD). X-ray diffraction data were collected on an X-ray diffractometer with Cu Kα radiation (Smartlab, Rigaku, Tokyo, Japan). The mineral phases were identified using the vendor-provided software (Smartlab Studio) with ICDD’s PDF-2 as the reference database. The phases were identified first by automated identification and then manual peak matching with the references constrained by elemental composition.

### 2.4 Synchrotron X-ray Absorption Spectroscopy

We used X-ray absorption spectroscopy to identify and quantify the species of Ca and K. The X-ray absorption near-edge structure (XANES) spectra at the K-edge of Ca and K were collected at the soft X-ray micro characterization beamline (SXRMB) at the 8BM beamline (TES) at the National Synchrotron Light Source II (NSLS, Upton, New York).

The biomass and ash samples (fine powders) were spread as a thin and homogeneous film on a carbon tape (attached to a sample holder) and mounted on a sample holder. The sample holder was mounted in a sample chamber under a helium atmosphere. The beam size (unfocused) was 2 mm × 5 μm. The Ca K-edge XANES spectra were acquired at the energy range from 4020 to 4200 eV and the energy steps were: 2.15 eV for 4020 to 4035 eV, 0.3 eV for 4035 to 4070 eV, 1 eV for 4070 to 4130 eV, and 2.5 eV for 4130 to 4200 eV. The K K-edge XANES spectra were acquired at the energy range from 3582 to 3682 eV and the energy steps were: 2 eV for 3582 to 3607 eV, 0.3 eV for 3607 to 3647 eV, and 1 eV for 3647 to 3682 eV. At least two scans were collected for each sample. The Ca K-edge spectra of hydroxyapatite were collected along with each batch of samples for postcollection energy calibration, by calibrating the pre-edge peak at 4040 eV.^15^

The spectra were merged, background subtracted, and normalized before linear combination fitting (LCF). All analyses were performed using the Athena package.^28^ Speciation of Ca and K in the biomass and burned samples was estimated by using LCF of the sample spectrum with spectra of reference compounds. The reference compounds were first identified based on common minerals identified in biomass ash via XRD,^16,29^ and then the ones used for LCF were selected based on principle component analysis (PCA) and target transformation. Sources of the reference compounds and their spectra can be found in Table S1 and Figure S1.

### 2.5 Ash Dissolution Experiments

Release kinetics experiment: three ash samples (SC550, ROL550, and ROL750) from two biomasses and two temperatures that differed the most in Ca speciation were selected for dissolution experiments. The ash samples were mixed with deionized water at a ratio of 300 mg per 200 mL deionized water without pH adjustment. The solutions were continuously stirred on a stir plate. Without adjustment, the pH was highly alkaline and stayed at 11–12. The composition of the aqueous phase was monitored over a period of 30 h. Specifically, ∼3 mL of solution was sampled at 0.5, 1, 2, 5, 8, 12, and 30 h after mixing by a syringe and then filtered through a 0.45 μm syringe filter.

Dissolution experiment at varying pHs: two ash samples and two prescribed fire residues were mixed with deionized water, and pH was adjusted twice to 4.0, 6.0, and 8.0 after 1 and 8 h of mixing, using HCl and NaOH. The ash samples were mixed at a ratio of 45 mg/30 mL, and the fire residues were mixed at a ratio of 200 mg/30 mL, in 50 mL centrifuge tubes. The tubes were placed in a shaker and agitated at 150 rpm for 30 h, and 5 mL of solution was sampled and filtered through a 0.45 μm syringe filter. The pH at the time of sampling was recorded and reported because it remained relatively stable (ΔpH < 0.15) beyond 30 h. Concentrations of Ca, Mg, K, Na, and P in the filtered solutions were measured by diluting the filtered solutions in 2% HNO_3_ and analysis using ICP-OES. Two internal standards (ytterbium and scandium) were added to the standards and samples to correct for potential matrix effects or instrument sensitivity fluctuation.

The systems were also simulated with PHREEQC (PHREEQC Interactive, V3.0),^30^ to determine the chemical species (aqueous and precipitated species) at varying pH conditions (Text S1).

## 3 Results and Discussion

### 3.1 Physicochemical Properties of Charcoal and Ash

The elemental composition of the biomass and some ash samples (550 °C) has been reported and is included herein with additional samples (Table S2).^26^ The biomass has Ca/P molar ratios ranging from 1.7 to 238 and Ca/K ratios ranging from 0.4 to 26, covering the molar ratios of biomass in many terrestrial ecosystems such as boreal and temperate forests and savannas.^6,31^ The sample set enables evaluation of the effects of biomass composition on Ca speciation. The two prescribed fire residues consisted of significant amounts of volatile matter (22 and 33 wt %), indicating the presence of incomplete burning material.

Significant differences in the mineralogy existed among ash samples from different biomass and temperatures (Figure S2). Overall, ROL ash and APBP_Wood showed strong diffraction patterns that indicate the presence of highly crystalline phases, while SC ash and APBP_Grass showed relatively weak diffraction peaks and contained amorphous phases. The main mineral phases identified in all ash samples were calcite, quartz, and hydroxyapatite (not identifiable in prescribed fire samples), and MgO, CaO, and Ca hydroxide were identified only in 750 °C ash samples. The dominance of calcite, quartz, and hydroxyapatite diffraction peaks was also observed in the ash of spruce, pine, and aspen woods.^32^ The relative intensity of hydroxyapatite diffraction was greater in SC samples than in ROL samples and was greater at 750 °C than at 550 °C, corresponding to the relatively large Ca/P ratio in SC and increasing crystallinity of hydroxyapatite as temperature increased.^26,33^ The presence of oxides only at 750 °C corresponds to the formation condition of these phases (e.g., above 600 °C for CaO).^34^

### 3.2 Transformation of Ca Speciation during Incomplete and Complete Burning

**Figure 1:**
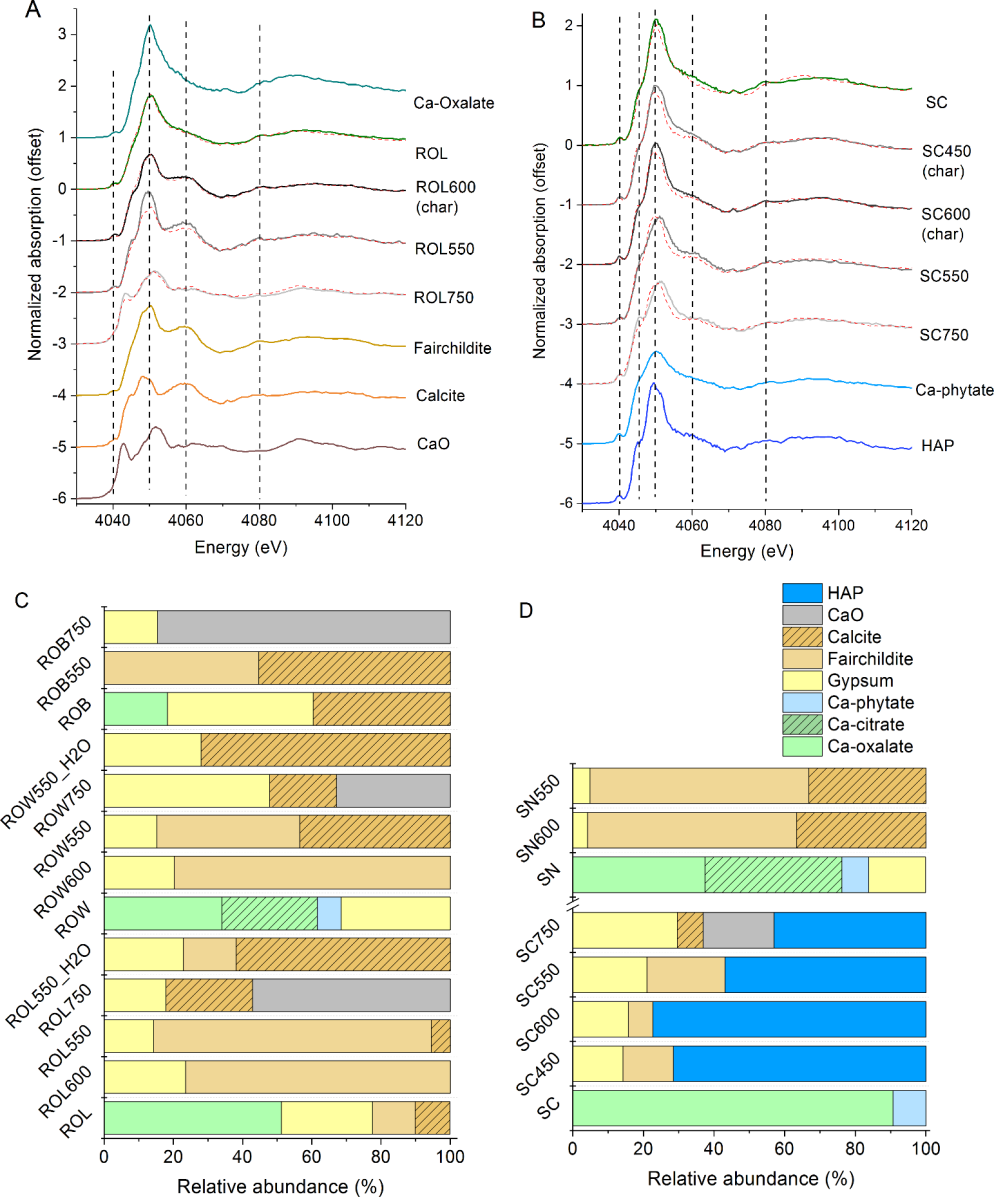
Calcium K-edge XANES spectra for charcoal and ash for (A) red oak leaf (ROL) and (B) spruce cone (SC), as well as reference compounds. Red dash lines are the fits. Calcium speciation for red oak (C) and Norway spruce (D) samples estimated by LCF of the Ca K-edge XANES spectra. The samples were labeled as abbreviation + temperature, with ROL, ROW, and ROB being biomass of red oak leaf, red oak wood, and red oak bark, respectively. SC and SN are Norway spruce cone and needle biomass, respectively. Overall, the LCF uncertainty was <10%, except for a few cases such as ROW, ROB550, and hydroxyapatite for SC samples.

Quantitative speciation of Ca in the biomass and ash samples was achieved by using Ca K-edge XANES analysis. As shown in the Ca K-edge XANES spectra, heating induced temperature-dependent changes to each of the biomass samples, while the changes differed among the biomass samples, especially between spruce cone and others (Figure 1A,B and Figure S3). For biomass with relatively high Ca/P molar ratios (i.e., leaf, bark, and wood), the most significant changes occurred at absorption at about 4042, 4050, and 4060 eV that correspond to the spectral features of carbonates and oxides. For the spruce cone and its thermal processed products, changes at 4046, 4050, and 4060 eV were the most significant, which correspond to the spectral feature of hydroxyapatite.

Calcium speciation from LCF of the spectra shows that Ca existed predominantly as organic complexes in biomass (>50%, except for 18% for red oak bark), which were transformed into carbonates, phosphates, and/or sulfates at intermediate heating temperature (∼550 °C), then Ca oxide at higher temperature (Figure 1 and Tables S3 and S4). Relative abundances of these mineral phases differ between biomass samples that differ in elemental stoichiometry (Ca/P molar ratio). For ash of biomass with large Ca/P ratios (7.8 to 238) that was formed at 550 °C, Ca existed predominantly as fairchildite (about 40–80%), calcite (5–55%), and gypsum (∼15%) at lower abundances (Figure 1C). As the temperature increased to 750 °C, the main change was the formation of CaO and the disappearance of fairchildite. The larger the Ca/P ratio (7.8 to 238), the more CaO formation (33–85%) at 750 °C. Depending on the plant parts, calcite and gypsum may also be present at varying abundances (15–50%) at 750 °C. In contrast, ash of spruce cone (Ca/P ≈ 2) at 550 °C was dominated by hydroxyapatite (∼60%) and consisted of ∼20% of fairchildite and gypsum. At 750 °C, hydroxyapatite and gypsum remained stable, while fairchildite was replaced by calcite and CaO (Figure 1D). In general, the speciation result agrees with the XRD data and thermal phase transition of Ca minerals.^34^ For example, K–Ca double carbonates follow a transition order of butschliite–fairchildite – K_2_Ca_2_(CO_3_)_3_, as temperature increases to about 700 °C, which are decomposed into oxides as temperature increases further.^35^

Calcium speciation for charcoal produced at 450 and 600 °C was also characterized. Despite the difference in oxygen level, Ca speciation for charcoal was similar to that of ash formed at 550 °C. This suggests that the transformation of Ca during fire burning depends mainly on temperature but not on O_2_. Following the hydration of the ash formed at 550 °C, the main change was the replacement of fairchildite by calcite (Figure 1C), corresponding to the release of potassium into the aqueous phase (Section 3.2).

It is worth noting that LCF of Ca K-edge XANES spectra possess uncertainties, especially for species lacking strong spectral features, such as different organic complexes, gypsum, and hydroxyapatite (reflected in large fitting errors of some biomass samples and chemical species, Tables S3 and S4). This is similar to the case of LCF of P K-edge XANES data, in which the fitting result can deviate 30 to 50% from the actual abundance for species lacking spectral features.^36^ Therefore, the LCF-estimated speciation result shall be interpreted semiquantitatively. However, this uncertainty does not compromise the overall findings of the thermal transformation of Ca speciation and the effects of elemental stoichiometry.

### 3.3 Speciation of Macronutrients in Prescribed Fire Residues

**Figure 2:**
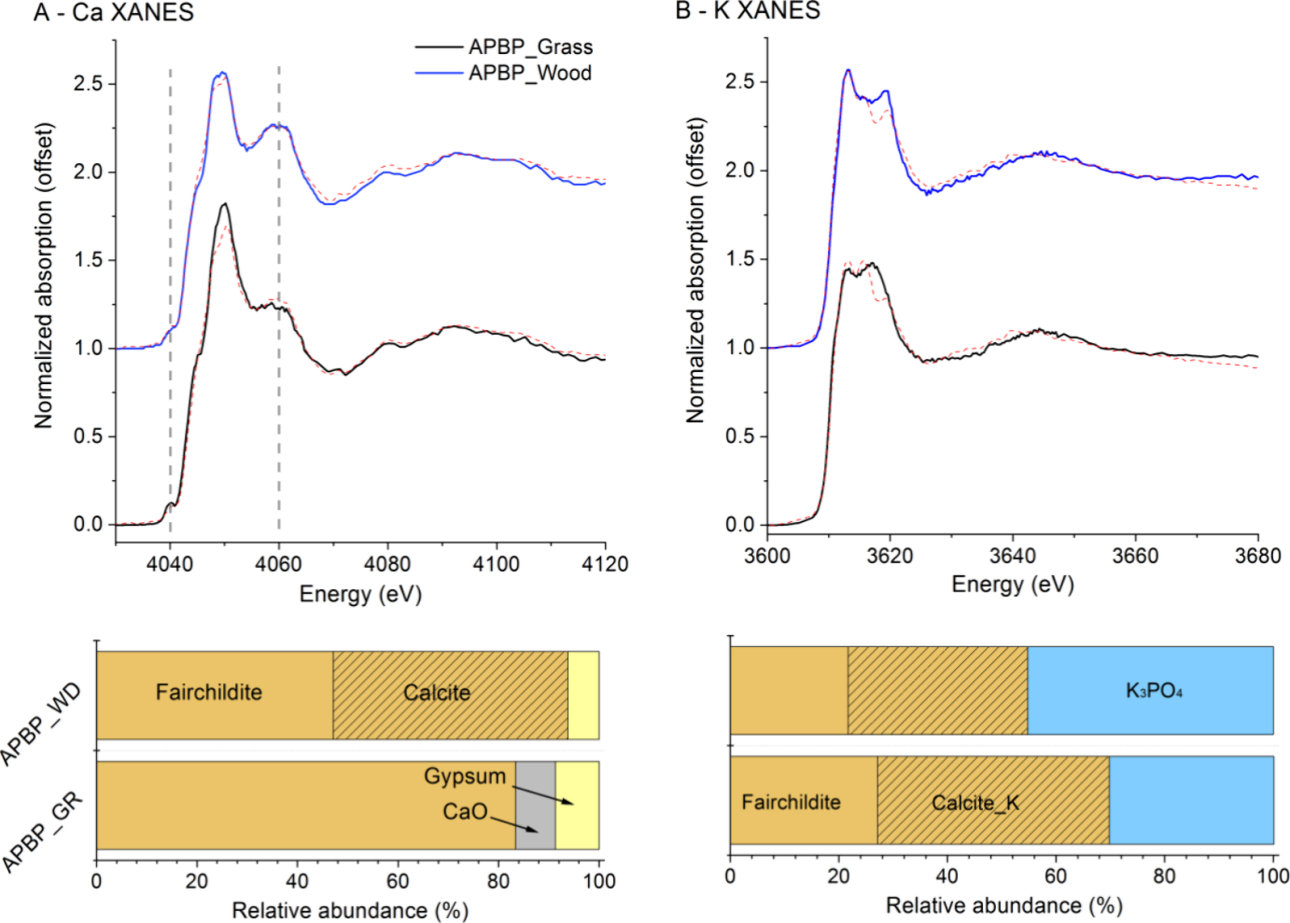
K-edge XANES spectra and LCF results for Ca (A) and K (B) in two prescribed fire residues. Red dash lines are the fits.

Prescribed fire residues consisted of similar Ca species identified in ash produced from uniform biomass at controlled thermal conditions, while differences existed between the two samples (Figure 2). APBP_Grass consisted of dominant fairchildite (∼80%) and small amounts of Ca oxide and gypsum, while APBP_Wood contained similar amounts of fairchildite and calcite, as well as a small fraction of gypsum. Potassium speciation confirmed the presence of fairchildite (Ca–K double carbonates) and K-doped calcite (trace amount of K), as well as K phosphates. The two samples mainly differed in the relative abundance of these K species. The observed difference in Ca and K speciation is possibly the result of different fuel compositions and burning behaviors. For example, Ca speciation of the APBP_Wood sample was similar to ash of biomass samples such as SN550, ROB550, and ROW550 that have a large Ca/P ratio (>7.8) and are main litter sources for a forest. The high abundance of fairchildite in APBP_Grass agrees with its relatively small Ca/K molar ratio (2.1) compared to that of APBP_Wood (7.0). The result manifests the effects of fuel biomass composition on fire ash composition and elemental speciation, and biomass macronutrient stoichiometry (Ca:K:Mg:P) generally varies broadly across ecosystems.^6,31^

Ca oxide was not identified in these prescribed fire residues, except for a limited quantity in the grass-prescribed fire residue. This suggests that the prescribed fire temperature did not reach 750 °C during the prescribed fires, consistent with common temperature profiles (maximum temperature ranged between 150 to 600 °C) recorded during prescribed fires.^37,38^

### 3.4 Mineral Dissolution and Macronutrient Release

**Figure 3:**
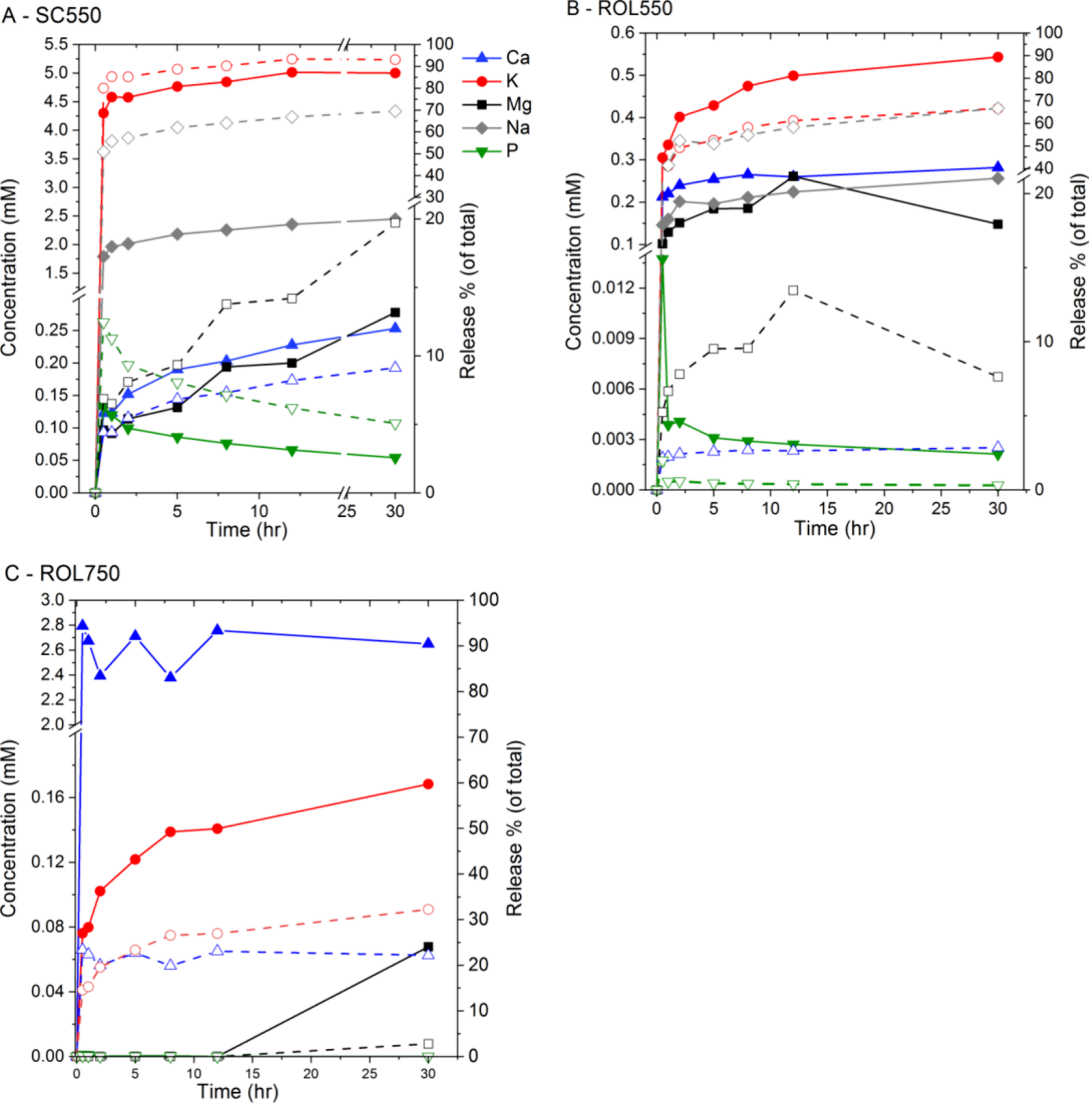
Dissolution rates of macronutrients from three ash samples: SC550 (A), ROL550 (B), and ROL750 (C) in deionized water (pH remained stable at 11.3 ± 0.1). The data were expressed as both solution concentration (filled symbols and solid line) and percentage of total content (empty symbol and dash line).

**Figure 4:**
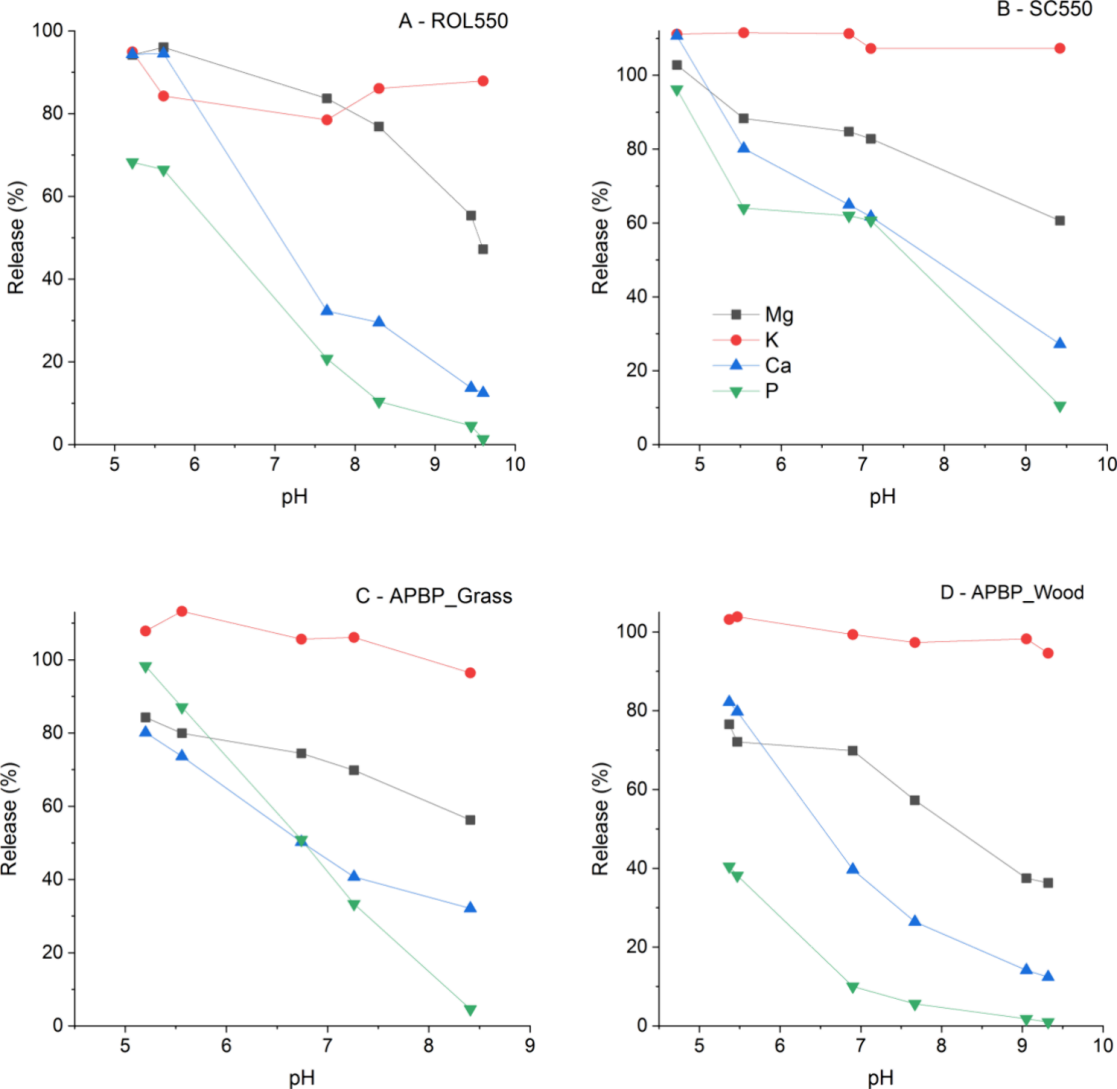
Release of four macronutrients (Mg, K, Ca, and P) from complete combustion ash of red oak leaf (A, ROL550) and spruce cone (B, SC550) and from prescribed fire residues (C, APBP_Grass and D, APBP_Wood) at pH between 5 and 9.5, after 30 h.

Batch experiments showed that macronutrients were differentially dissolved into the aqueous phase, while the rates and extents depended on biomass composition and pH. For ash of spruce cone and red oak leaf generated at 550 °C (SC550 and ROL550, respectively), the dissolution kinetics differed between macronutrients and samples (Figure 3). Both Na and K were rapidly released within the initial 2 h and after that, the release slowed down. The release rates of Ca and Mg for the same ash were similar and were different between the two ash samples. Both Ca and Mg were rapidly released within only the initial 2 h for ROL ash, while were gradually released during the 30-h experiment for SC550. Phosphorus was quickly released for the first time point (30 min), while its concentration decreased gradually, possibly caused by phosphate precipitation following Ca dissolution. Solubility (% of total) of the macronutrients followed the order K ≅ Na > Mg > Ca > P and increased with decreasing pH (Figure 4). Significant differences in the extent of dissolution existed between ash samples. At pH 11, the solubility (both concentration and %) of all macronutrients in SC550 was much higher than those in ROL550. For example, 95% of total K and 70% of total Na in SC550 were dissolved, compared to about 70% of total K and total Na in ROL550. Similarly, ∼ 20% and 10% of Mg and Ca can be respectively released from the SC ash, compared to only ∼10% and 4% for ROL550. The water solubility of P was relatively limited, with ∼5% (or 0.05 mM) P being soluble for SC550 and <1% (2.5 μM) P for the ROL550. For ROL ash formed at high temperatures (ROL750), Ca became more soluble, with ∼20% of total Ca (∼2.6 mM) being dissolved. However, the solubility of other macronutrients (K, Mg, and P) was significantly reduced. The increase of Ca solubility corresponds to the formation of CaO at 750 °C, which is transformed into hydroxides after hydration and is more soluble than carbonates. The reduced Mg solubility corresponds to the formation of MgO (as shown by XRD) that is poorly soluble in water.

Dissolution of K, Ca, Mg, and P from the tested ash showed different pH dependences (Figure 4). The dissolution of K showed no pH dependency and the rest decreased as pH increased. Comparatively, both Ca and P were highly sensitive to pH and their solubility increased sharply as pH decreased from 9 to 5. Comparatively, Mg was less sensitive and remained highly soluble at pH above 7. Equilibrium calculation showed that the systems were over-saturated for calcite hydromagnesite, and hydroxyapatite (saturation index ∼0) at alkaline pH and for only hydroxyapatite as pH decreased below 9, resulting in the pH-dependent dissolution of Ca, P, and Mg (Figure S4). Less P was dissolved from ROL550 than from SC550, consistent with the experimental results and the relatively high Ca/P ratio of ROL550.

Overall, the macronutrient dissolution behavior of prescribed fire residues was similar to that of completely burned ash despite the different thermal conditions under which they formed. The main difference is that the extent of macronutrient release of prescribed fire samples was not as complete as the ash samples because the prescribed fire samples consisted of incomplete burned materials that physically bound part of the macronutrients (Table S2).

### 3.5 Interplay between Biomass Composition and Thermal Conditions in Controlling the Speciation and Solubility of Macronutrients

By characterizing a thermosequence of char and ash samples, we were able to reveal the thermochemical transformation of Ca across fire thermal conditions: (1) organic complexes of Ca were transformed into various inorganic species that were primarily controlled by temperature and were less affected by the oxygen level; (2) elemental stoichiometry regulates the relative abundances of different inorganic Ca species, such as the dependency of the abundance of Ca carbonates and phosphates and Ca–K double carbonates (fairchildite) on Ca/P and Ca/K ratios; and (3) Ca carbonates undergo phase transformation that depends on temperature (Figure 1). Although previous studies have revealed the main Ca species biomass burned ash by XRD,^16,29^ this study further quantified the abundance of these species and revealed the effect of biomass composition and heating temperature, using uniform biomass with varying elemental stoichiometries and X-ray absorption spectroscopy. The Ca speciation data agrees with P speciation data characterized for the same sample set, in which Ca phosphate was identified as the main P species in ash and charcoal, particularly for samples from biomass with large Ca/P molar ratios (>5).^26^ The findings highlight the importance of biomass chemical composition in controlling wildland fire ash composition, especially considering the variation of vegetation types and macronutrient stoichiometry among ecosystems.^6^ In fact, the elemental composition of wildland fire ash was shown to be highly variable,^39^ it is expected that chemical speciation of macronutrients (including Ca) will also differ.

The speciation data correspond to the aqueous solubilization behaviors of the macronutrients. First, the predominant existence of Ca and P as Ca carbonates and hydroxyapatites corresponded to the pH-dependent dissolution of these two macronutrients. In particular, Ca and P were closely associated and Ca regulates the solubility of P. For example, P in ash with a large Ca/P ratio existed predominantly as crystalline apatite and thus was less soluble than P in ash with a small Ca/P ratio, in which P formed significant soluble P species other than apatite (Figures 3 and 4). In addition, the solubilization of Ca also can regulate the solubility of P, since the soluble Ca and P may reprecipitate (Figure 3). For K, it existed primarily as Ca–K double carbonates or phosphates in ash and thus was highly soluble across a wide pH range. For example, fairchildite was transformed into calcite after ash was hydrated (Figure 1), corresponding to the solubilization of K at pH = 11. The pH-dependency of macronutrient solubilization and variation among macronutrients suggest that fire-derived macronutrients will be differently cycled, affecting their retention and soil availability in the burned ecosystems.^40,41^

This study also revealed the effect of fire thermal condition on Ca speciation and aqueous solubilization behavior of macronutrients. Mineralization of macronutrients during fire was affected more by temperature than by O_2_ level, as shown in similar speciation of Ca and P in ash and charcoal at similar temperature ranges (Figure 1).^26,42^ However, burning completeness affects the extent of nutrient release as a result of the matrix effect. For example, although the order and pH dependency of nutrient dissolution of prescribed fire residues were similar to those of completely burned ash, less macronutrients (except for K) were released as a result of physical constraints in incomplete burned carbonaceous components (Figure 4).^42^

## 4 Environmental Implications

Fire represents the most devastating disturbance to many terrestrial ecosystems, changing or even resetting critical ecosystem structures and functions. The cycling of macronutrients is among the many functions that are disturbed by fires, and the transformation of biomass into fire residues represents the direct and main impact of fires. This study is the first to quantitatively characterize the thermochemical behavior of Ca during biomass burning and relate it to the aqueous solubilization behavior of macronutrients, which is fundamental for exploring postfire cycling dynamics of macronutrients.

The mechanistic understanding of the interplay between biomass composition and thermal condition in controlling the speciation Ca in fire residues can possibly be generalized across terrestrial ecosystems that differ in plant composition and fire severity with the used approach (varying biomass composition and broad thermal conditions). The results pinpoint the importance of fuel sources (stoichiometric contribution of plant species and compartments) in controlling fire residue chemistry, specifically macronutrient chemistry. With current efforts devoted to understanding the disturbance of fires to ecosystems and their succession, it is important to account for the composition and stoichiometric contribution of fuel biomass of a burned ecosystem in addition to fire fuel loads and fire severity.

Because Ca is of pedogenic and ecological significance and generally is the most abundant element in fire residues, the improved understanding of Ca chemistry in fire residues can help predict its postfire geochemical behavior. In particular, soil macronutrient content and availability in terrestrial ecosystems rely heavily on the recycling of aboveground biomass pools, which are mineralized during fire. The quantitative speciation and solubilization results from this study can help predict the pathways and rate of Ca (and other macronutrients) returning to soils.

The obtained fundamental chemical data and understanding will be of interest to many disciplines, such as forest ecology, ecosystem management, and water science, which study the fate and transport of wildland fire ash.

## Supporting Information Available

Elemental composition of the tested ash samples; XRD patterns of ash and prescribed fire residue samples; sources and normalized K-edge XANES spectra of the reference compounds and burned samples, and linear combination fitting results; PhreeQC calculation of macronutrient solubility (PDF)
