# Supplementary material for: Thermochemical Transformation of Calcium during Biomass Burning and the Effects on Postfire Aqueous Dissolution of Macronutrients
Source: Environ Sci Technol. 2024 Sep 18;58(39):17304–12. doi: 10.1021/acs.est.4c04820 (PMC11447901; doi:10.1021/acs.est.4c04820)
Supplement: Supplementary file 1 — es4c04820_si_001.pdf [file es4c04820_si_001.pdf]

Supporting Information

Thermochemical transformation of calcium during biomass burning and the effects on post-fire aqueous dissolution of macronutrients

Rixiang Huang[1*], Sarah Nicholas[2] and Zheng Wei[3]

1. Department of Environmental and Sustainable Engineering, University at Albany
1400 Washington Ave, Albany, New York, 12222, USA

2. National Synchrotron Light Source II, Brookhaven National Laboratory,
Upton, NY 11793, USA

3. Department of Chemistry, University at Albany
1400 Washington Ave, Albany, New York, 12222, USA

*Corresponding Author:

Email: rhuang6@albany.edu

Phone: 518-437-4977

Numbers of pages: 10

Numbers of Tables: 4

Numbers of Figures: 4

Text S1. Equilibrium modeling using PHREEQC

Chemical speciation in the dissolution experiments (varying pH) was simulated using PHREEQC (PHREEQC Interactive, V3.0) - a low temperature aqueous geochemical calculations computer program [1]. The batch reaction mode and the minteq.V4 thermodynamic database distributed with the software were used. The main input parameters for the simulation include concentrations of major elements (entered in the liquid phase in its atomic form), solution pH, electric potential (*pe*), and temperature. Concentrations of Ca, Mg, K, Na, P, S, Cl, and C (as CO_3^{2-}) were based on the ash/water ratio and elemental concentrations of the ash. A range of carbonates, hydroxides, phosphates, and sulfates of Ca and Mg were selected as the equilibrium phases. The input pH changed from 12 to 8, which resulted in final pH ranged from 11.8 to ~6.0.

The initial amount of all equilibrium phases was set to zero. All simulations were performed at a temperature of 25 °C and 1 atm of atmosphere. Aqueous concentration of Ca, Mg, and P presented were plot against pH.

Table S1. Reference compounds used for linear combination fitting (LCF) of Ca and K K-edge XANES data.

Compound	Source	Ref.
Calcium oxalate (monohydrate)	Thermo Scientific Chemicals (99%)	
Calcium acetate	Thermo Scientific Chemicals (ACS Reagent)	
Calcium citrate (tribasic tetrahydrate)	Thermo Scientific Chemicals (98+%)	
Ca phytate	Titration of 0.1 M $CaCl_2$ into 0.1 M phytic acid at pH 8.0. The precipitates were filtered and air-dried	2
Fairchildite	Equal moles of K_2CO_3 and $CaCO_3$ heated at 920 K in CO_2 atmosphere for 72 hours.	3
Calcite	Eisco mineral specimen	
Ca oxide (lime)	Honeywell Fluka (Puriss grade)	
Hydroxyapatite (HAP)	Preheated (90°C) 100 mL 0.1 mol/L $Ca(CH_3COO)_2$ and 100 mL 0.06M $(NH_4)_2HPO_4$ were mixed and aged at 90 °C for 2 days (mixed solution pH = 5.0), then washed and filtered.	4
Gypsum	Eisco mineral specimen	
Potassium phosphate	Thermo Scientific Chemicals (Reagent grade)	
Potassium doped calcite	Synthetic calcite doped with K	5

Table S2. Selected elemental composition of ash samples of different plant parts.

Biomass	Label	T (°C)	TC (%)	Ca/P	Ca/K	Content (wt%)				
						P	K	Ca	Mg	Na
Spruce cone	SC550	550	-	2.6	0.5	2.20	13.97	7.40	2.25	5.40
	SC750	750	-	1.6	0.4	4.19	20.34	8.59	3.58	7.52
**Spruce needle**	**SN550**	**550**	**-**	**7.8**	**2.0**	**1.26**	**6.02**	**12.6**	**0.81**	**0.94**
Red oak leaf	**ROL550**	**550**	**-**	**16.4**	**7.6**	**0.98**	**2.66**	**20.73**	**2.42**	**0.61**
	ROL750	750	-	15.6	22.8	1.58	1.36	31.86	3.88	1.10
**Red oak wood**	**ROW550**	**550**	**-**	**9.2**	**1.7**	**1.50**	**10.30**	**17.94**	**1.76**	**0.67**
**Red oak bark**	**ROB550**	**550**	**-**	**238**	**26.4**	**0.13**	**1.47**	**39.92**	**0.31**	**0.37**
Prescribed fire residues	APBP_GR	Grass	22	4.6	2.1	1.00	2.75	5.89	1.04	0.12
	APBP_WD	Wood	33	14.8	7.0	0.55	1.47	10.48	0.91	0.16

Note: data of red oak leaf, wood, and bark ash samples at 550 °C (Bold) were from Wu *et al*. (2023)[6]. Total C content (TC) was determined as weight loss following ignition (550 °C for 4 hours).

Table S3. LCF analysis results for Ca K-edge XANES spectra of red oak samples.

Sample	R-factor	Ca Speciation (%)						
		Ca-oxalate	Ca-citrate	Ca-phytate	Gypsum	Fairchildite	Calcite	CaO
ROL	0.0046	51.2 (3.5)	0	0	26.3 (3.6)	12.4 (5.5)	10 (2.4)	0
ROL600	0.0051	0	0	0	23.5 (2.1)	76.5 (3.3)	0	0
ROL550	0.0155	0	0	0	14.3 (3.7)	80.2 (4.6)	5.5 (2.8)	0
ROL750	0.0192	0	0	0	17.8 (2.4)	0	25.1 (2.9)	57.1 (4.7)
ROL550_H2O	0.0200	0	0	0	22.9 (8.0)	15.2 (5.2)	61.9 (5.0)	0
ROW	0.0176	34 (12.5)	27.6 (18.9)	6.9 (1.5)	31.5 (31.3)	0	0	0
ROW600	0.0079	0	0	0	20.2 (3.6)	79.8 (2.3)	0	0
ROW550	0.0028	0	0	0	15.2 (1.3)	41.3 (1.6)	43.5 (2.1)	0
ROW750	0.0050	0	0	0	47.8 (1.9)	0	19.3 (2.2)	32.9 (4.5)
ROW550_H2O	0.0195	0	0	0	28.0 (3.3)	0	72.0 (7.4)	0
ROB	0.0211	18.3 (1.6)	0	0	42.1 (2.2)	0	39.6 (6.0)	0
ROB550	0.0053	0	0	0	0	44.7 (15.7)	55.3 (3.7)	0
ROB750	0.0115	0	0	0	15.4 (3.0)	0	0	84.6 (1.9)

Table S4. LCF analysis results for Ca K-edge XANES spectra of Norway spruce samples.

Sample	R-factor	Ca Speciation (%)							
		Ca-oxalate	Ca-citrate	Ca-phytate	Gypsum	Fairchildite	Calcite	CaO	HAP
SC	0.0110	90.7 (2.6)	0	9.3 (5.3)	0	0	0	0	0
SC450	0.0152	0	0	0	14.2 (4.7)	14.3 (6.5)	0	0	71.5 (11.0)
SC600	0.0107	0	0	0	15.7 (3.9)	7.0 (5.5)	0	0	77.3 (9.9)
SC550	0.0231	0	0	0	21.0 (5.4)	22.1 (10.9)	0	0	56.9 (13.6)
SC750	0.0247	0	0	0	29.6 (8.1)	0	7.3 (7.7)	20.1 (4.0)	43.0 (16.8)
SN	0.0032	37.4 (2.2)	38.8 (4.6)	7.6 (2.4)	16.1 (3.2)	0	0	0	0
SN600	0.0053	0	0	0	4.2 (3.9)	59.2 (5.4)	36.6 (15.1)	0	0
SN550	0.0048	0	0	0	4.9 (1.7)	61.9 (5.8)	33.2 (1.6)	0	0

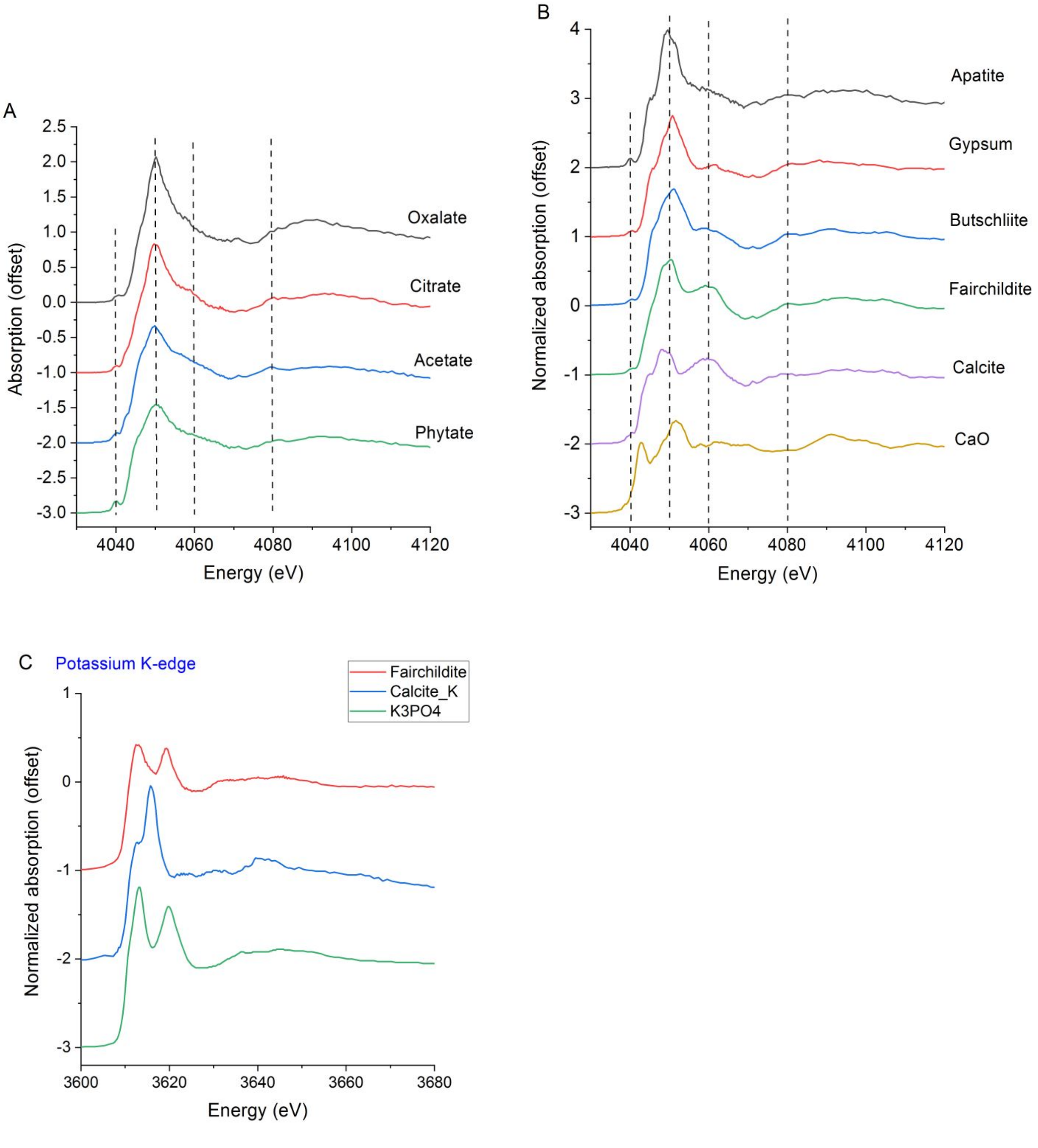

Figure S1. Calcium K-edge XANES spectra for Ca organic complexes (A) and Ca minerals (B). Potassium K-edge XANES spectra for the used references (C).

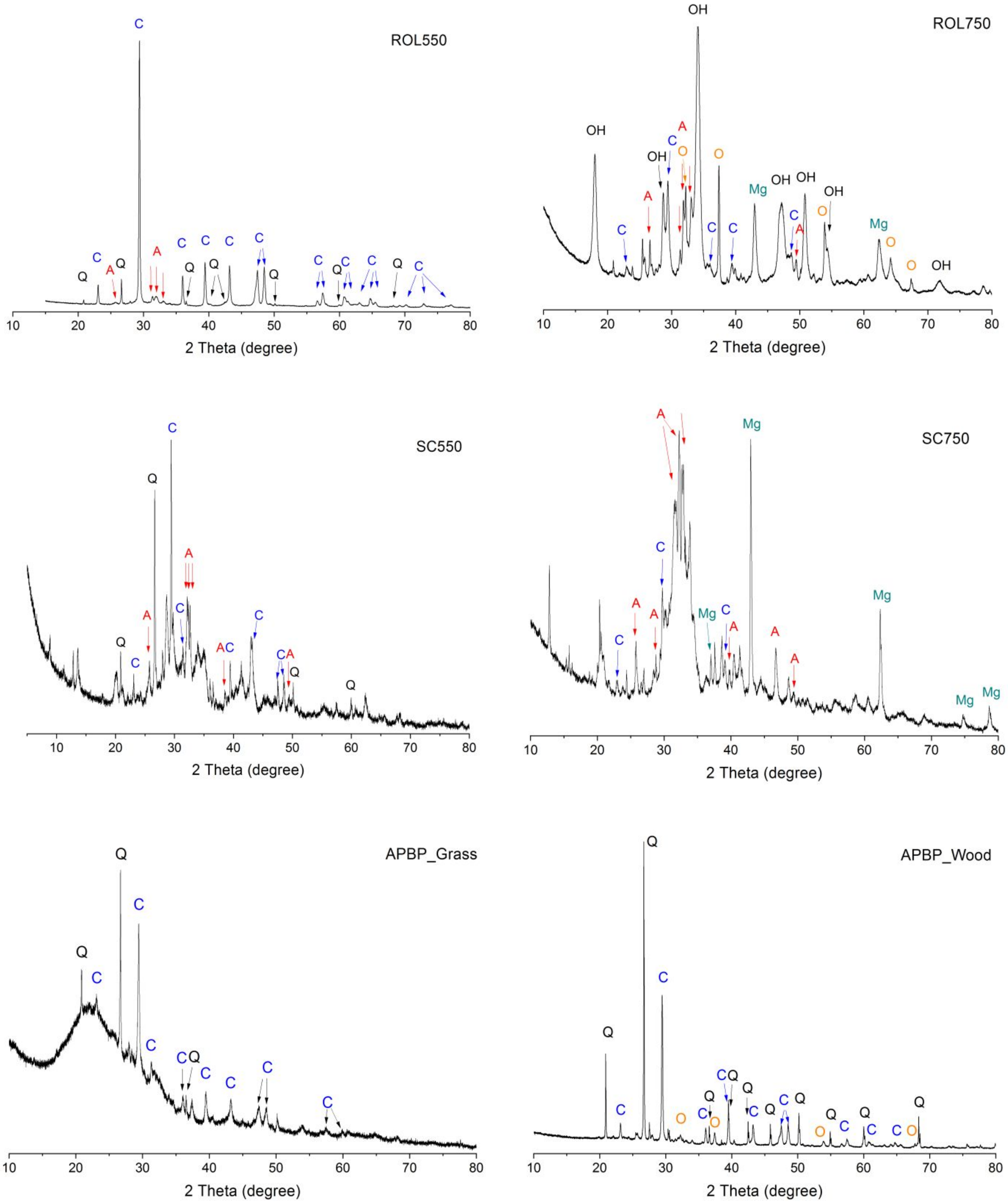

Figure S2. XRD data of the ash samples (SC550, SC750, ROL550, ROL750, APBP-grass, APBP-wood). A – hydroxyapatite, C – calcite, Mg –MgO (periclase), O – CaO, OH – Ca hydroxide, Q – quartz.

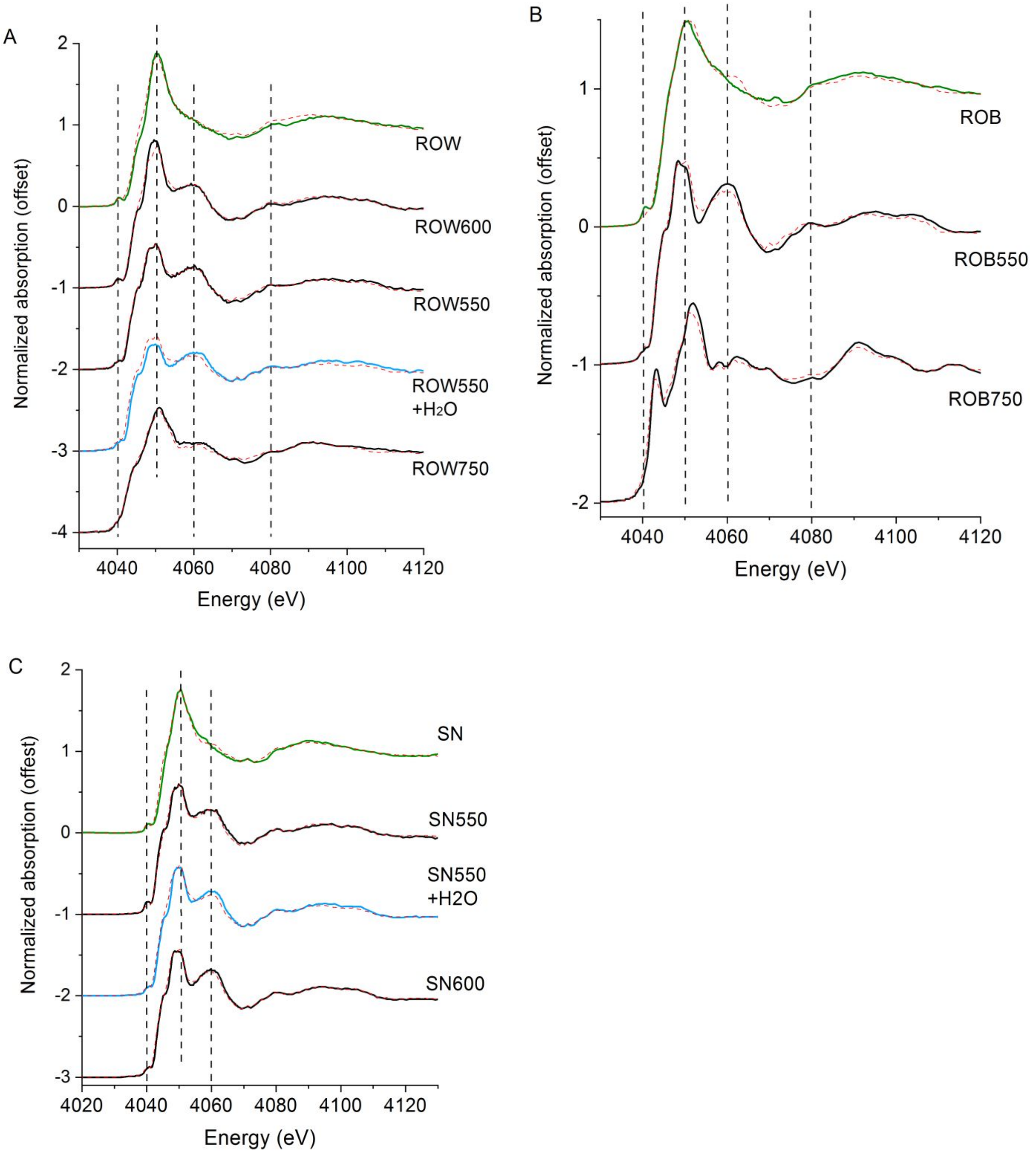

Figure S3. Calcium K-edge XANES spectra for raw biomass, char, and ash samples of red oak wood (A), red oak bark (B), and Norway spruce needle (C). Red dash lines were the fits.

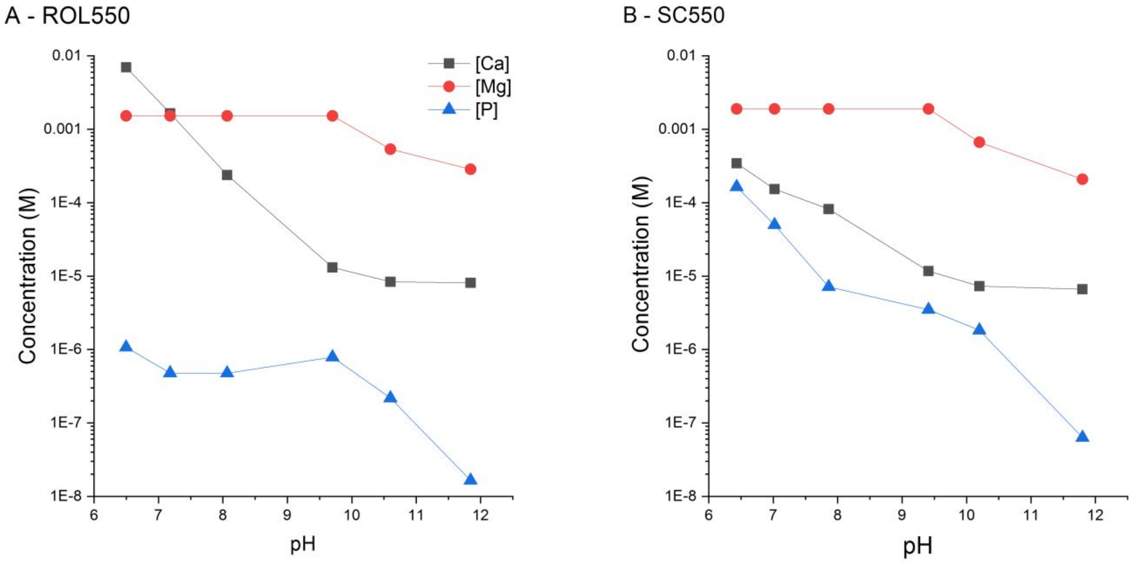

Figure S4. Thermodynamic calculation (phase equilibrium) of aqueous concentrations of Ca, Mg, and P of ROL550 and SC550 ash samples in water, based on the same conditions for the dissolution experiments (Fig. 4).

References

1. Parkhurst, D. L.; Appelo, C. *Description of input and examples for PHREEQC version 3: a computer program for speciation, batch-reaction, one-dimensional transport, and inverse geochemical calculations*; 2328-7055; US Geological Survey: 2013.
2. Grynspan, F.; Cheryan, M., Calcium phytate: effect of pH and molar ratio on in vitro solubility. *Journal of the American Oil Chemists' Society* **1983,** *60*, (10), 1761-1764.
3. Navrotsky, A.; Putnam, R. L.; Winbo, C.; Rosén, E., Thermochemistry of double carbonates in the K2CO3-CaCO3 system. *American Mineralogist* **1997,** *82*, (5-6), 546-548.
4. Huang, R. X.; Fang, C.; Zhang, B.; Tang, Y. Z., Transformations of Phosphorus Speciation during (Hydro)thermal Treatments of Animal Manures. *Environ Sci Technol* **2018,** *52*, (5), 3016-3026.
5. Li, W.; Liu, X.-M.; Hu, Y., Potassium and Calcium K-Edge XANES in Chemical Compounds and Minerals: Implications for Geological Phase Identification. *Geostandards and Geoanalytical Research* **2020,** *44*, (4), 805-819.
6. Wu, Y.; Pae, L. M.; Gu, C.; Huang, R., Phosphorus Chemistry in Plant Ash: Examining the Variation across Plant Species and Compartments. *ACS Earth and Space Chemistry* **2023,** *7*, (11), 2205-2213.
